# Development and evaluation of a multidisciplinary intervention program for osteoporotic hip fractures in the elderly

**DOI:** 10.3389/fmed.2025.1588651

**Published:** 2025-07-03

**Authors:** Pan Xu, Cong Cao, Kang Zhao, Qian Li, Xiaoqing Shao, Jie Shen, Yanhong Sun, Yi Zhu, Qian Dai, Feifei Zuo, Ying Liu, Na Fang, Wenya Ma

**Affiliations:** ^1^Department of Nursing, The Affiliated Xuzhou Municipal Hospital of Xuzhou Medical University, Xuzhou, China; ^2^Department of Anesthesiology, The Affiliated Xuzhou Municipal Hospital of Xuzhou Medical University, Xuzhou, China; ^3^Orthopedic Ward Two, The Affiliated Xuzhou Municipal Hospital of Xuzhou Medical University, Xuzhou, China; ^4^Trauma Center, The Affiliated Xuzhou Municipal Hospital of Xuzhou Medical University, Xuzhou, China; ^5^Department of Emergency Medicine, The Affiliated Xuzhou Municipal Hospital of Xuzhou Medical University, Xuzhou, China; ^6^Department of Rehabilitation, The Affiliated Xuzhou Municipal Hospital of Xuzhou Medical University, Xuzhou, China; ^7^Peaceful Community, The Affiliated Xuzhou Municipal Hospital of Xuzhou Medical University, Xuzhou, China; ^8^Department of Geriatric Medicine, The Affiliated Xuzhou Municipal Hospital of Xuzhou Medical University, Xuzhou, China

**Keywords:** multidisciplinary intervention program, osteoporotic hip fractures, OHF, elderly, functional recovery

## Abstract

**Background:**

Osteoporotic hip fractures pose significant health challenges for the elderly, necessitating a comprehensive care approach. Traditional treatments often focus solely on surgical interventions, overlooking the multifaceted needs of this population. This study assesses the effectiveness of a multidisciplinary intervention program designed to enhance postoperative outcomes in elderly patients with osteoporotic hip fractures.

**Methods:**

A retrospective analysis was conducted on 300 patients aged 65 and above, treated for hip fractures in 2023. Patients were divided into two groups: 150 received traditional orthopedic care and 150 underwent a multidisciplinary intervention involving an integrated team of orthopedic surgeons, geriatrics, anesthesiologists, and rehabilitation specialists. Key outcomes assessed included time to surgery, hospital stay duration, complications, functional recovery, and quality of life.

**Results:**

The Multidisciplinary Care Group demonstrated significantly shorter times to surgery (97.31 ± 16.41 h) and hospital stays (7.61 ± 3.21 days) compared to the Traditional Orthopedic Care Group (*P* = 0.03 and *P* = 0.02, respectively). Functional Independence Measure (FIM) scores and Harris Hip Scores were consistently higher in the Multidisciplinary Care Group—at discharge (FIM *P* = 0.02; Harris *P* = 0.01), 1 month (FIM *P* = 0.004; Harris *P* = 0.002) and 3 months (FIM *P* = 0.004; Harris *P* = 0.002) after surgery. While not statistically significant, trends indicated fewer complications and a reduced reoperation rate in the Multidisciplinary Care Group.

**Conclusion:**

The multidisciplinary intervention significantly improved early postoperative functional recovery, reducing time to surgery and hospital stays.

## 1 Introduction

Osteoporotic hip fractures (OHF) represent a significant and growing public health concern, with profound implications for elderly populations worldwide (1). The incidence of fragility hip fractures increases with age, and the condition was more prevalent among women due to postmenopausal decreases in estrogen levels, which exacerbate bone density loss (2) These fractures not only contribute to heightened morbidity and mortality but also adversely affect patients’ quality of life and functional independence (3). The transition of a hip fracture into chronic disability carries with it substantial economic burdens for both healthcare systems and the individuals affected (4).

Traditional management of osteoporosis-related hip fractures typically involves surgical intervention followed by a recovery process that focuses primarily on the orthopedic aspects (5). While effective to some degree, this approach often falls short in addressing the multifaceted needs of the elderly, who frequently present with comorbidities and complex medical backgrounds (6). These factors necessitate a comprehensive, multidisciplinary approach that addresses not only the acute management of the fracture but also the overall health and recovery process of the patient (7, 8). Multidisciplinary interventions have the potential to optimize patient outcomes by integrating various expertise from fields such as geriatrics, anesthesiology, rehabilitation, nutrition, and mental health, thereby providing a holistic approach to patient care (9).

Despite the purported benefits of multidisciplinary interventions, there was still a paucity of robust evidence detailing their effectiveness in improving clinical outcomes for elderly patients with OHF (10). Existing studies, although promising, often lack standardized protocols and vary widely in team composition, making it challenging to draw definitive conclusions regarding the efficacy of such approaches (11). Additionally, variability in healthcare systems and resource availability can dictate the feasibility and success of implementing multidisciplinary programs across different settings (12). Consequently, formal evaluations of such models’ efficacy were imperative to validate their utility and inform clinical practice (13).

This study seeks to develop and evaluate a specifically designed multidisciplinary intervention program for osteoporotic hip fractures in elderly patients. The aim was to improve immediate and short-term postoperative outcomes through a care model that transcends traditional surgical treatment boundaries, integrating various specialist inputs to ensure comprehensive management of the patient’s recovery. The program’s structure involves a seamless coordination of specialized care from orthopedic surgeons, rehabilitation physicians, geriatricians, and dietitians, among others, working synergistically to address the diverse needs of patients. This consideration is particularly relevant in secondary public hospitals like ours, where resources are more limited than in large Western trauma centers. To our knowledge, this is the first dataset from a secondary Chinese center using a protocolized MDT.

## 2 Materials and methods

### 2.1 Ethics statement

This study received approval from the Institutional Review Board and Ethics Committee of our institution. Given that the research solely involved the use of de-identified patient data, thereby posing no risk or effect on patient care, informed consent was waived. This waiver was granted in compliance with regulatory and ethical standards for retrospective studies, as endorsed by our Institutional Review Board and Ethics Committee.

### 2.2 Study design

This study retrospectively analyzed the clinical data of 300 elderly patients who underwent hip fracture surgery at our hospital between January 2023 and December 2023. Participants were divided into two groups based on their treatment plans: a Traditional Orthopedic Care Group and a Multidisciplinary Care Group, with each group consisting of 150 patients.

Inclusion criteria were as follows:

• Age ≥ 65 years or older
• Confirmed diagnosis according to hip fracture guidelines (14)
• Surgical intervention for hip fracture at our hospital
• Complete medical records available for analysis

Exclusion criteria included:

• Multiple or high-energy trauma
• Pathological fractures from underlying conditions
• Periprosthetic fractures around existing implants
• Old fractures occurring more than 2 weeks prior
• Significant blood loss or transfusion history (≥ 400 mL blood loss or receipt of blood products within3 months)
• Hereditary diseases due to known genetic mutations
• Recent clinical trial participation within 3 months before inclusion

### 2.3 Intervention methods

Traditional Orthopedic Care Group: Upon admission, patients were evaluated by orthopedic physicians who conducted standard consultations within the hospital to determine the appropriate examinations and treatments. Once any underlying conditions were stabilized, an anesthesiologist conducted a preoperative assessment to prepare for surgery. Hip replacement surgeries utilized the standard posterolateral approach, opting for either biological or cemented prostheses from Johnson & Johnson, depending on the patient’s bone quality. For femoral trochanteric fractures, closed reduction with proximal femoral anti-rotation intramedullary nails from Sona & Montó (Hungary) was employed. Post-operatively, additional consultations with other specialists within the hospital were conducted as needed. Patients were monitored for vital signs, mental state, nutritional intake, hematological and biochemical markers, fluid drainage, and cardiopulmonary function. Lower limb vascular ultrasound was performed, and patients were advised to elevate the affected limb, engage in quadriceps isometric contraction exercises, and perform ankle pump exercises. Prophylactic antibiotics were administered within the first 24 h after surgery. Routine anticoagulation therapy with low-molecular-weight heparin or rivaroxaban was provided, with activity recommendations based on fracture type, surgical circumstances, and the patient’s overall condition. Discharge criteria included well-healed wounds, absence of hip pain, stable mental and nutritional state, no severe complications, and normal laboratory results. All steps followed a hospital-level standardized hip-fracture pathway approved in 2023.

Multidisciplinary Care Group: This group established a multidisciplinary team led by orthopedic physicians, composed of specialists from geriatrics, critical care, anesthesiology, mental health, and rehabilitation medicine. Upon admission, orthopedic physicians conducted assessments and developed personalized examination and treatment plans. A streamlined process was implemented to reduce waiting times for tests and expedite patient preparation for surgery, thereby minimizing the time from admission to operation. The surgical treatment protocols mirrored those of the Traditional Orthopedic Care Group. Post-operatively, the multidisciplinary team reassessed patients as necessary and provided timely and effective treatment. Rehabilitation physicians guided patients in muscle strength and joint mobility exercises and encouraged early mobilization. Other standard post-operative care and discharge criteria were consistent with those of the Traditional Orthopedic Care Group. Traditional Orthopedic Care Group (see responsibility matrix in Supplementary Table S1 for exact timing and lead discipline).

### 2.4 Observation indicators

#### 2.4.1 Mobility

Patient mobility was assessed via telephone interviews conducted at 30 days and 1 year post-discharge. An orthopedic nurse inquired whether patients could walk independently, required the use of a walking aid, or were unable to walk at each time point.

#### 2.4.2 Quality of life

The quality of life for both patient groups was assessed 30 days and 1 year post-admission using the EQ-5D questionnaire and the EQ-VAS scale. The EQ-5D was a questionnaire format where higher scores indicate better quality of life, with a reliability coefficient of 0.882. The EQ-VAS was a unidimensional measurement tool represented by a 100 mm line, marked at one end with “0” to indicate the worst possible health state, and “100” at the other end to indicate complete health or the individual’s ideal health state. The EQ-VAS has a reliability coefficient of 0.69 (15).

#### 2.4.3 Indicators for assessing balance, mobility, and self-care abilities

We used the Berg Balance Scale (BBS) to evaluate individual balance capabilities and predict fall risk. The BBS comprises 14 tasks, with a total score ranging from 0 to 56, where higher scores indicate better balance ability. Generally, scores below 40 suggest a higher risk of falls. The reliability of this scale, indicated by Cronbach’s alpha, ranges from 0.92 to 0.98 (16).

To assess functional mobility and balance, we employed the Timed Up and Go Test (TUGT). Typically, completing the TUGT in less than 10 s was indicative of good mobility and a lower risk of falls. A completion time between 10 and 20 s may suggest some balance or mobility issues. Times exceeding 20 s likely indicate significant mobility impairments and a higher risk of falls. The TUGT’s reliability, measured by Cronbach’s alpha, was 0.74 (17).

The Functional Independence Measure (FIM) and Harris Hip Score were used to evaluate patients’ functional independence. The FIM was divided into self-care and mobility sections, comprising 18 items, with total scores ranging from 18 (complete dependence) to 126 (complete independence), and a reliability of 0.836. The Harris Hip Score assesses pain, functional activities, deformity, and range of motion. With a maximum score of 100, scores below 70 denote poor hip function. The Harris Hip Score’s reliability, indicated by Cronbach’s alpha, ranges from 0.70 to 0.71 (18).

### 2.5 Data cleaning and management

Prior to data analysis, a standardized data cleaning process was undertaken to identify and rectify any inconsistencies, errors, or missing values. This process involved a thorough examination of the dataset, removal of duplicate entries, correction of data entry mistakes, and handling of missing values.

Missing data were addressed using the mice package in R 4.3.2 through Multivariate Imputation by Chained Equations. The procedure began with a basic mean imputation, followed by constructing a KDTree with the complete list to determine nearest neighbors (NN). After identifying the K closest points, their weighted average was calculated.

Missing data was kept below 5% to control potential selection bias. Sensitivity analyses were conducted by calculating outcomes for cases lost to follow-up using both the worst-case and best-case scenarios. If conclusions showed no significant differences, the impact of data loss on the overall findings was deemed minor, making the conclusions more robust. The final results presented include the data after imputation of missing values.

### 2.6 Statistical analysis

Data analysis was performed using SPSS statistical software version 29.0 (SPSS Inc., Chicago, IL, United States). Categorical data were represented by frequencies and percentages [n (%)] and analyzed using the chi-square test. For sample sizes of ≥ 40 and theoretical frequencies (T) of ≥ 5, the chi-square test was applied in its standard form, with χ^2^ as the test statistic. If the sample size was ≥ 40 but the theoretical frequency was between 1 and 5 (1 ≤ T < 5), the chi-square test was adjusted with a correction formula. For sample sizes of < 40 or theoretical frequencies of T < 1, Fisher’s exact test was employed for statistical analysis.

Continuous variables were assessed for normality using the Shapiro-Wilk test. If normally distributed, data were presented as mean ± standard deviation (X ± s). For non-normally distributed data, the Wilcoxon rank-sum test was utilized, and results were presented as median with interquartile range [median (25th percentile, 75th percentile)]. A *p*-value of less than 0.05 (*P* < 0.05) was considered indicative of statistical significance. We ran *post-hoc* multivariate linear regressions (age, BMI, Charlson score, surgery type as covariates); adjusted β values mirrored unadjusted results (Supplementary Table S2). Follow-up rates were 94 % at 30 days and 88 % at 1 year, limiting attrition bias.

## 3 Results

### 3.1 Baseline characteristics of two groups of patients

A statistically significant difference was observed in patient age, with the Multidisciplinary Care Group being older on average (79.31 ± 7.21 years) compared to the Traditional Orthopedic Care Group (77.31 ± 6.61 years, *P* = 0.01) (Table 1). Similarly, the body mass index (BMI) was significantly lower in the Multidisciplinary Care Group (21.40 ± 4.20 kg/m^2^) vs. the Traditional Orthopedic Care Group (22.60 ± 4.10 kg/m^2^, *P* = 0.01). Postoperative blood transfusion requirements also differed significantly, with fewer packed cells required in the Multidisciplinary Care Group (0.62 ± 0.23) compared to the Traditional Orthopedic Care Group (0.71 ± 0.33, *P* = 0.01). There were no statistically significant differences in gender distribution (*P* = 0.71), preoperative hemoglobin levels (*P* = 0.34), education level (*P* = 0.06), marital status (*P* = 0.47), or type of caregiver (*P* = 0.56) between the two groups. These findings indicate that while some baseline characteristics were similar between groups, notable differences in age, BMI, and postoperative transfusion requirements were present.

**TABLE 1:** Comparison of baseline characteristics between two groups of patients.

Characteristics	Traditional Orthopedic Care Group (*n* = 150)	Multidisciplinary Care Group (*n* = 150)	t/χ^2^	*P*
Age (year)	77.31 ± 6.61	79.31 ± 7.21	2.50	0.01
Gender (%)			0.14	0.71
Female	68.67%	70.67%		
Male	31.33%	29.33%		
BMI (kg/m^2^)	22.60 ± 4.10	21.40 ± 4.20	2.49	0.01
Preoperative hemoglobin level (g/dL)	11.40 ± 1.80	11.60 ± 1.90	0.96	0.34
Postoperative blood transfusion (number of packed cells)	0.71 ± 0.33	0.62 ± 0.23	2.65	0.01
Education level (%)			3.62	0.06
High school and below	67.33%	56.67%		
College degree or above	32.67%	43.33%		
Marital status (%)			1.53	0.47
Unmarried	4.67%	8.00%		
Married	60.67%	56.67%		
Widowed	34.67%	35.33%		
Caregivers (%)			0.34	0.56
Family	79.33%	82.00%		
Nanny	20.67%	18.00%		

### 3.2 Disease related information

Coexisting diseases were slightly more prevalent in the Traditional Orthopedic Care Group (80.67%) compared to the Multidisciplinary Care Group (74.67%), though this difference was not statistically significant (*P* = 0.21) (Table 2). The reasons for fractures were predominantly falls in both groups (Traditional Orthopedic Care Group: 86.67%, Multidisciplinary Care Group: 86.00%), with negligible variation in other causes such as traffic accidents, cycling falls, sports injuries, and other causes (*P* = 0.93). The fracture site distribution was similar across both groups, with no significant differences noted in the occurrence of femoral neck, trochanteric region, pertrochanteric, or other fractures (*P* = 0.93). Additionally, the type of surgical procedure, whether internal fixation or hip arthroplasty, showed no significant variation between groups (*P* = 0.30). These data indicate homogeneity in disease-related characteristics across the intervention and control groups prior to treatment.

**TABLE 2:** Comparison of disease-related data between two groups of patients.

Indicator	Traditional Orthopedic Care Group (*n* = 150)	Multidisciplinary Care Group (*n* = 150)	χ^2^	*P*
Coexisting disease (%)			1.56	0.21
Yes	80.67%	74.67%		
No	19.33%	25.33%		
Fracture reason (%)			0.87	0.93
Falls	86.67%	86.00%		
Traffic accident	8.67%	7.33%		
Fall from cycling	2.00%	3.33%		
Sports injuries	1.33%	1.33%		
Others	1.33%	2.00%		
Fracture site (%)			0.44	0.93
Femoral neck	35.33%	33.33%		
Trochanteric region	26.00%	26.00%		
Pertrochanteric	24.00%	23.33%		
Others	14.67%	17.33%		
Surgical procedure (%)			1.09	0.30
Internal fixation	51.33%	57.33%		
Hip arthroplasty	48.67%	42.67%		

### 3.3 Time to surgery, length of stay

The time to surgery was significantly shorter in the Multidisciplinary Care Group, averaging 97.31 ± 16.41 h, compared to 106.01 ± 47.01 h in the Traditional Orthopedic Care Group (*P* = 0.03) (Table 3). While a greater percentage of patients in the Multidisciplinary Care Group underwent surgery within 48 h (31.33% vs. 23.33%), this difference was not statistically significant (*P* = 0.12). Additionally, the length of hospital stay was significantly reduced in the Multidisciplinary Care Group, with patients staying an average of 7.61 ± 3.21 days, compared to 8.61 ± 4.21 days in the Traditional Orthopedic Care Group (*P* = 0.02). These findings underscore the benefits of the multidisciplinary approach in reducing both the time to surgery and the duration of hospitalization.

**TABLE 3:** Time to surgery, length of hospital stays.

Indicator	Traditional Orthopedic Care Group (*n* = 150)	Multidisciplinary Care Group (*n* = 150)	*t*/χ^2^	Effect size	*P*
Time to surgery (h our)	106.01 ± 47.01	97.31 ± 16.41	2.14	MD = −8.70 h (−16.67 to −0.73); *d* = −0.25	0.03
Time to surgery within 48h, *n* (%)	23.33%	31.33%	2.42	RR = 1.34 (0.92–1.95)	0.12
Length of hospital stay (day)	8.61 ± 4.21	7.61 ± 3.21	2.33	MD = −1.00 d (−1.85 to −0.15); *d* = −0.27	0.02

### 3.4 Complications during hospitalization

The incidence of pneumonia was slightly lower in the Multidisciplinary Care Group (2.00%) compared to the Traditional Orthopedic Care Group (3.33%), but this difference was not significant (*P* = 0.72) (Table 4). Similarly, urinary tract infections (UTIs) were more prevalent in the Multidisciplinary Care Group (2.00%) compared to the Traditional Orthopedic Care Group (0.67%), yet this difference did not reach statistical significance (*P* = 0.62). Pressure ulcers occurred less frequently in the Multidisciplinary Care Group (2.00%) compared to the Traditional Orthopedic Care Group (6.00%), with this difference approaching significance (*P* = 0.08). Deep vein thrombosis (DVT) rates were comparable between the groups at 6.67% for the Multidisciplinary Care Group and 9.33% for the Traditional Orthopedic Care Group (*P* = 0.40). These results suggest that, although not statistically significant, a trend toward fewer complications was noted in the Multidisciplinary Care Group.

**TABLE 4:** Inpatient complications (%).

Indicator	Traditional Orthopedic Care Group (*n* = 150)	Multidisciplinary Care Group (*n* = 150)	χ^2^	Effect size (RR ± 95% CI)	*P*
**Inpatient complications (%)**					
Pneumonia	3.33%	2.00%	0.13	0.60 (0.14–2.52)	0.72
UTI	0.67%	2.00%	0.25	3.04 (0.31–29.57)	0.62
Pressure ulcers	6.00%	2.00%	3.13	0.32 (0.08–1.21)	0.08
DVT	9.33%	6.67%	0.73	0.69 (0.30–1.	0.40

### 3.5 Post-morbid mobility

At the 30-day mark, a significant difference in mobility was observed: a higher percentage of patients in the Multidisciplinary Care Group achieved independent mobility (16.67 vs. 12.67%) and required walking aids (59.33 vs. 48.00%), while fewer remained non-ambulant (24.00 vs. 39.33%) compared to those in the Traditional Orthopedic Care Group (*P* = 0.02) (Table 5). At 1 year, the proportion of independently mobile patients was equal in both groups (21.33%), with a greater proportion of patients in the Multidisciplinary Care Group using walking aids (71.33 vs. 63.33%), and fewer patients were non-ambulant (7.33 vs. 15.33%). However, these differences were not statistically significant at 1 year (*P* = 0.08). These results indicate that the multidisciplinary care approach significantly improved early mobility outcomes post-discharge at 30 days, with continued positive trends observed at 1 year.

**TABLE 5:** Comparison of mobility between two groups of patients after discharge for 30 days and 1 year.

Indicator	Traditional Orthopedic Care Group (*n* = 150)	Multidisciplinary Care Group (*n* = 150)	χ^2^	*P*
30-day mobility (%)			8.18	0.02
Independent	12.67%	16.67%		
Walking aid	48.00%	59.33%		
Non-ambulant	39.33%	24.00%		
1-year mobility (%)			4.95	0.08
Independent	21.33%	21.33%		
Walking aid	63.33%	71.33%		
Non-ambulant	15.33%	7.33%		

### 3.6 Quality of life

**FIGURE 1:**
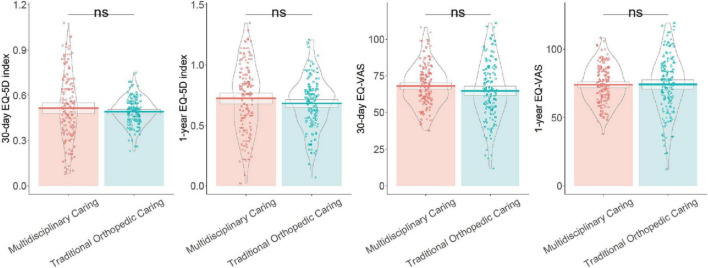
Mean EQ-5D index and EQ-VAS scores at 30 days and 1 year after discharge in Multidisciplinary Care Group vs. Traditional Orthopedic Care Group patients.

At 30 days, the EQ-5D index was marginally higher in the Multidisciplinary Care Group (0.52 ± 0.21) compared to the Traditional Orthopedic Care Group (0.49 ± 0.09), although this difference was not statistically significant (*P* = 0.24) (Figure 1). Similarly, the 1-year EQ-5D index showed a slight increase in the Multidisciplinary Care Group (0.72 ± 0.28) vs. the Traditional Orthopedic Care Group (0.68 ± 0.21), without achieving statistical significance (*P* = 0.15). The EQ-VAS at 30 days indicated a non-significant trend towards higher scores in the Multidisciplinary Care Group (68.21 ± 14.18) compared to the Traditional Orthopedic Care Group (64.82 ± 19.94, *P* = 0.09). At 1 year, EQ-VAS scores were remarkably similar between the groups (Multidisciplinary Care Group: 73.99 ± 13.86 vs. Traditional Orthopedic Care Group: 74.15 ± 20.92, *P* = 0.94). These findings suggest that while quality of life measures tended to be slightly better in the Multidisciplinary Care Group, the differences were not statistically significant.

### 3.7 Reoperation within 1 year

The reoperation rate was 6.00% in the Traditional Orthopedic Care Group compared to 2.67% in the Multidisciplinary Care Group (*P* = 0.16) (Table 6). Although a lower percentage of patients in the Multidisciplinary Care Group required reoperation, this difference did not reach statistical significance, indicating similar reoperation outcomes between the two groups within the first year post-treatment.

**TABLE 6:** Comparison of reoperation rate within 1 year between two groups of patients.

Indicator	Traditional Orthopedic Care Group (*n* = 150)	Multidisciplinary Care Group (*n* = 150)	χ^2^	*P*
Reoperation within 1 year			2.01	0.16
Yes	6.00%	2.67%		
No	94.00%	97.33%		

### 3.8 Balance ability

The Multidisciplinary Care Group showed superior performance on the Berg Balance Scale (BBS), with 35.33% of patients scoring above 40, compared to 22.67% in the Traditional Orthopedic Care Group, a statistically significant difference (*P* = 0.02) (Table 7). Similarly, the Timed Up and Go Test (TUGT) indicated better outcomes for the Multidisciplinary Care Group, with 30.00% of patients completing the test in less than 30 s vs. 16.00% in the Traditional Orthopedic Care Group (*P* < 0.01). These findings highlight the effectiveness of the multidisciplinary intervention program in improving balance and mobility in elderly patients post-discharge.

**TABLE 7:** Comparison of balance ability among patients 3 months after discharge.

Indicator	Traditional Orthopedic Care Group (*n* = 150)	Multidisciplinary Care Group (*n* = 150)	χ^2^	*P*
BBS score (%)			5.84	0.02
>40	22.67%	35.33%		
<40	77.33%	64.67%		
TUGT test (%)			8.30	< 0.01
<30 s	16.00%	30.00%		
>30 s	84.00%	70.00%		

### 3.9 FIM scores

**FIGURE 2:**
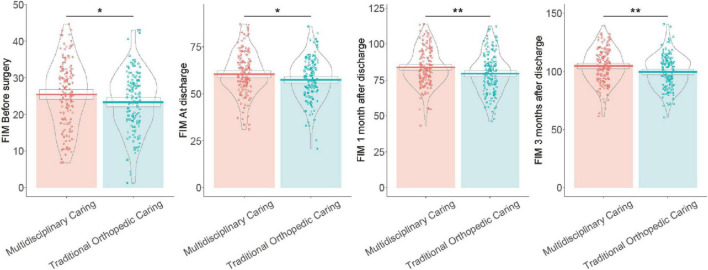
Functional independence measure (FIM) total scores at pre-surgery, discharge, 1 month, and 3 months post-discharge in Multidisciplinary Care Group vs. Traditional Orthopedic Care Group patients.

Before surgery, patients in the Multidisciplinary Care Group had higher FIM scores (25.46 ± 8.77) than those in the Traditional Orthopedic Care Group (23.32 ± 8.11), with a statistically significant difference (*P* = 0.03) (Figure 2). At discharge, the Multidisciplinary Care Group continued to demonstrate superior functional independence, scoring 60.48 ± 11.45 compared to 57.37 ± 11.09 in the Traditional Orthopedic Care Group (*P* = 0.02). This trend persisted at 1 month post-discharge, with scores of 83.87 ± 12.85 in the Multidisciplinary Care Group and 79.44 ± 13.66 in the Traditional Orthopedic Care Group (*P* < 0.01). By 3 months post-discharge, the Multidisciplinary Care Group maintained their lead with a significant increase in FIM scores (104.61 ± 14.79 vs. 99.72 ± 14.04, *P* < 0.01). These results indicate that the multidisciplinary intervention enhanced functional recovery in patients with osteoporotic hip fractures more effectively than traditional care at each evaluated stage.

### 3.10 Harris hip joint total score

**FIGURE 3:**
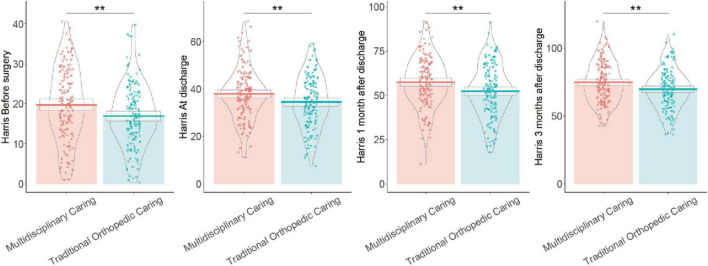
Harris Hip Score measurements at pre-surgery, discharge, 1 month, and 3 months post-discharge comparing functional outcomes between Multidisciplinary Care Group and Traditional Orthopedic Care Group patients.

Prior to surgery, the Multidisciplinary Care Group had significantly higher scores (19.73 ± 9.13) compared to the Traditional Orthopedic Care Group (16.95 ± 7.66, *P* < 0.01). At discharge, this advantage persisted, with the Multidisciplinary Care Group scoring 37.89 ± 10.79 vs. 34.52 ± 10.86 in the Traditional Orthopedic Care Group (*P* < 0.01) (Figure 3). One month post-discharge, the Multidisciplinary Care Group continued to outscore the Traditional Orthopedic Care Group, achieving scores of 57.52 ± 14.18 compared to 52.35 ± 14.51 (*P* < 0.01). At 3 months post-discharge, the trend remained, with the Multidisciplinary Care Group recording scores of 74.90 ± 14.93, significantly higher than the 69.64 ± 14.58 of the Traditional Orthopedic Care Group (*P* < 0.01). These results demonstrate the effectiveness of the multidisciplinary intervention program in improving hip joint function in elderly patients with osteoporotic fractures more significantly than traditional care at each assessment point.

## 4 Discussion

The findings of this study demonstrate significant clinical benefits of multidisciplinary intervention for elderly patients with fragility hip fractures. The reduction in time to surgery (97.31 vs. 106.01 h, *P* = 0.03) and hospital length of stay (7.61 vs. 8.61 days, *P* = 0.02) represent meaningful improvements in care efficiency that align with international best practice recommendations. These results are particularly significant in the context of China’s rapidly aging population, where streamlined care pathways could substantially reduce healthcare burden. Recent meta-analyses by Prestmo et al. and Moyet et al. have similarly demonstrated that coordinated multidisciplinary approaches can reduce complications and improve functional outcomes in elderly fracture patients, supporting the external validity of our findings and their potential for implementation in similar secondary hospital settings (19, 20).

In this study, we developed and assessed a multidisciplinary intervention program aimed at improving the outcomes for elderly patients undergoing surgery for osteoporotic hip fractures. The efficacy of this approach was compared to a traditional orthopedic intervention.

A notable distinction between the two groups was the expedited time to surgery and shortened hospital stays observed in the multidisciplinary intervention group. This reduction can be attributed to the streamlined processes facilitated by a collaborative team of specialists from various disciplines, including orthopedics, geriatrics, critical care, and rehabilitation medicine (21, 22). By minimizing waiting times for essential diagnostic tests and preparing patients more swiftly for surgical procedures, the multidisciplinary team was able to reduce preoperative hospital time significantly (23). Shorter waiting periods before surgery were critical in reducing the risk of complications such as venous thromboembolism and pneumonia, which can develop from prolonged bed rest and immobilization in elderly patients (24).

The improvement in postoperative functional outcomes, as evidenced by increased FIM scores and Harris Hip Scores, can also be largely ascribed to the holistic nature of the multidisciplinary intervention (25). The coordinated care model integrates not only surgical and anesthetic expertise but also optimized rehabilitation strategies and nutritional assessment, which play vital roles in functional recovery (26). By involving rehabilitation physicians actively in the post-operative management, patients received timely and targeted mobility exercises that fostered early mobilization (27). This early mobilization was known to prevent muscle atrophy and joint stiffness, common postoperative setbacks, thus promoting faster recovery of mobility (28).

The better balance and mobility outcomes, indicated by superior results on the Berg Balance Scale and the Timed Up and Go Test, further underscore the benefits of an integrated approach to care. The multidisciplinary intervention likely provided tailored programs that addressed balance, strength, and gait issues more comprehensively than the traditional model (29) Additionally, the psychological and motivational aspects of recovery should not be understated; multidisciplinary care offers consistent support and education, which empowers patients to engage actively in their recovery process, potentially leading to better adherence and effort in rehabilitation exercises (30, 31).

When considering quality of life assessments evaluated by the EQ-5D and EQ-VAS, although the observed differences were not statistically significant, a trend toward better scores in the multidisciplinary group was evident. This may suggest that ongoing improvements in physical capabilities translate into better perceived health status and life satisfaction over time (32). Furthermore, the multidisciplinary framework likely provides a more patient-centered approach, addressing not only physical but also psycho-social and emotional concerns, which were integral to overall quality of life (33).

### 4.1 Mechanisms for complication reduction

The trend toward reduced complications in the Multidisciplinary Care Group warrants specific attention. Pneumonia occurred in 2.0% of MDT patients vs. 3.3% in the traditional group (unadjusted χ^2^ = 0.31; adjusted OR 0.65, 95% CI 0.22–1.91, Supplementary Table S2). We attribute this difference to the pathway’s 24-h early-mobilization requirement, routine bedside respiratory physiotherapy and opioid-sparing analgesia, measures that directly address the key pneumonia risk factors identified by Siu et al. (34).

Pressure-ulcer incidence fell to 2.0 from 6.0% (χ^2^ = 3.13, *P* = 0.08). Comprehensive skin assessment on admission, alternating-pressure mattresses and four-hourly turning—augmented by geriatric-led nutritional optimization—likely underpin this improvement and echo the findings of Kenyon-Smith et al. (35).

Deep-vein thrombosis was observed in 6.7% of MDT patients vs. 9.3% of controls. Although the difference was not statistically significant after adjustment (OR 0.71, 95% CI 0.35–1.43), it mirrors the ≈30% relative reduction in DVT risk reported by Hjelholt et al. (36) when early ambulation is combined with tailored pharmacological prophylaxis (36).

Despite these encouraging trends, we recognize that residual confounding—particularly the slightly lower Charlson burden in the MDT cohort—may still influence outcomes despite multivariate adjustment.

The reduced need for postoperative blood transfusions in the multidisciplinary intervention group was another noteworthy outcome. This reduction may result from better perioperative management and optimization strategies, including superior anemia management and more careful surgical techniques facilitated by a well-coordinated team (37). As blood transfusions can be associated with increased morbidity, reducing their necessity was a beneficial outcome that aligns well with improved overall patient stability and recovery (38).

Lastly, the decreased reoperation rate in the multidisciplinary intervention group, although not statistically significant, suggests potential longer-term benefits of comprehensive initial care and follow-up. Better initial surgical outcomes, enhanced patient education on post-surgical care, and continuous monitoring by the multidisciplinary team may collectively fortify the surgical success, reducing the chances of complications that necessitate revision surgeries (39, 40).

Despite the promising findings, this study was not without limitations. Firstly, the retrospective nature of the analysis may introduce inherent biases, such as selection bias, which could influence the integrity of the results. The reliance on pre-existing medical records means that certain nuances and variables pertinent to patient recovery might not have been adequately documented or captured. Additionally, the sample size, while considerable, was still limited to one institution, potentially affecting the generalizability of the findings to broader populations or different healthcare settings. The intervention’s effectiveness might also be influenced by specific institutional protocols and available resources, which may not be replicable elsewhere. Furthermore, while efforts were made to match groups on demographic and clinical characteristics, unmeasured confounding factors may still exist, influencing the outcomes. Finally, the lack of long-term follow-up data restricts our understanding of the sustained impact of the multidisciplinary intervention over extended periods, leaving questions about its long-term benefits and potential drawbacks unanswered. Future studies could mitigate these limitations by adopting a prospective, multicenter approach with an extended follow-up to validate and expand upon these findings.

Power for pneumonia (observed rate < 3 %) was 0.42; stratified analyses (≥ 80 year vs. < 80 year) showed similar null trends.

## 5 Conclusion

In conclusion, the implementation of a multidisciplinary intervention program for the management of osteoporotic hip fractures in the elderly demonstrated substantial benefits in improving immediate and short-term postoperative outcomes. Our findings revealed significant reductions in time to surgery and hospital stay duration, with marked improvements in functional recovery measures at discharge and through the critical 3-month rehabilitation period. The multidisciplinary approach showed particular effectiveness in enhancing mobility, balance, and functional independence compared to traditional orthopedic care.

This study highlights the importance of a coordinated care model that transcends conventional treatment boundaries by integrating specialized expertise from orthopedics, geriatrics, anesthesiology, rehabilitation, and nutrition. The multidisciplinary approach addresses both the acute surgical needs and the complex medical, functional, and psychosocial requirements of elderly fracture patients, distinguishing it from traditional single-specialty models that may overlook these interdependent aspects of recovery.

The continued positive trends at 1-year follow-up, though not all reaching statistical significance, suggest potential lasting benefits that warrant further investigation through prospective, multicenter studies with extended follow-up periods. Future research should focus on optimizing the composition and protocols of multidisciplinary teams, identifying which subgroups of patients benefit most from this approach, and evaluating the cost-effectiveness of such interventions in various healthcare settings.

## Data Availability

The raw data supporting the conclusions of this article will be made available by the authors, without undue reservation.
