# Supplementary material for: Development and evaluation of a multidisciplinary intervention program for osteoporotic hip fractures in the elderly
Source: Front Med (Lausanne). 2025 Jul 3;12:1588651. doi: 10.3389/fmed.2025.1588651 (PMC12267227; doi:10.3389/fmed.2025.1588651)
Supplement: Supplementary file 1 [file Table_1.docx]

# Supplementary Table S1. Multidisciplinary Responsibility Matrix

**Traditional Orthopedic Care Group**

Exact timing and lead discipline for each key task within the standardized hip‑fracture pathway.

| **Timepoint** | **Lead discipline** | **Key task** | **Frequency** | **Record method** |
| --- | --- | --- | --- | --- |
| Admission (≤ 6 h) | Orthopedic | Imaging + fasting order | Once | EMR |
| Pre‑op 24 h | Anesthesiology | ASA score + optimize nutrition | Once | SOAP note |
| Post‑op 24 h | Rehabilitation | Bedside mobility assessment | Daily | e‑rehab form |
| Post‑op 72 h | Nursing | Early ambulation + DVT prophylaxis | Daily | Nursing chart |
| Weekly until discharge | MDT Round | Review goals, adjust care plan | Weekly | MDT note |
| Follow‑up 30 d | Community nurse | Telephone FIM + EQ‑5D | Once | CRF |
