# Supplementary material for: Development and evaluation of a multidisciplinary intervention program for osteoporotic hip fractures in the elderly
Source: Front Med (Lausanne). 2025 Jul 3;12:1588651. doi: 10.3389/fmed.2025.1588651 (PMC12267227; doi:10.3389/fmed.2025.1588651)
Supplement: Supplementary file 2 [file Table_2.docx]

# Supplementary Table S2. Adjusted outcomes from multivariate linear regression

β coefficients adjusted for age, BMI, Charlson score, and surgery type.

| **Outcome** | **Adjusted β (95 % CI)** | **P-value** |
| --- | --- | --- |
| FIM at discharge | +3.1 (0.6–5.5) | 0.01 |
| Length of stay (days) | −1.1 (−1.8 to −0.3) | 0.006 |
| Pneumonia (logit OR) | −0.43 (−1.51 to 0.65) | 0.43 |
